# A Shape-Adaptive Gallic Acid Driven Multifunctional Adhesive Hydrogel Loaded with Scolopin2 for Wound Repair

**DOI:** 10.3390/ph15111422

**Published:** 2022-11-17

**Authors:** Huan Chen, Tingting Zheng, Chenyang Wu, Jinrui Wang, Fan Ye, Mengyao Cui, Shuhui Sun, Yun Zhang, Ying Li, Zhengqi Dong

**Affiliations:** 1 Drug Delivery Research Center, Institute of Medicinal Plant Development, Chines Academy of Medical Sciences, Peking Union Medical College, Beijing 100193, China; 2 Department of Pharmaceutics, School of Pharmaceutical Sciences, Hebei Medical University, Shijiazhuang 050017, China; 3 Department of Pharmaceutics, School of Pharmaceutical Sciences, Heilongjiang University of Chinese Medicine, Harbin 150036, China; 4 Key Laboratory of Bioactive Substances and Resources Utilization of Chinese Herbal Medicine, Ministry of Education, Chinese Academy of Medical Sciences, Peking Union Medical College, Beijing 100094, China; 5 Key Laboratory of New Drug Discovery Based on Classic Chinese Medicine Prescription, Beijing 100700, China; 6 Beijing Key Laboratory of Innovative Drug Discovery of Traditional Chinese Medicine (Natural Medicine) and Translational Medicine, Beijing 100700, China

**Keywords:** shape-adaptive adhesive hydrogel, gallic acid, scolopin2, wound healing, antioxidant, antibacterial

## Abstract

Wound healing is one of the major challenges in the biomedical fields. The conventional single drug treatment has unsatisfactory efficacy, and the drug delivery effectiveness is restricted by the short retention on the wound. Herein, we develop a multifunctional adhesive hydrogel that can realize robust adhesion, transdermal delivery, and combination therapy for wound healing. Multifunctional hydrogels (CS-GA-S) are mixed with chitosan-gallic acid (CS-GA), sodium periodate, and centipede peptide-scolopin2, which slowly releases scolopin2 in the layer of the dermis. The released scolopin2 induces the pro-angiogenesis of skin wounds and enables excellent antibacterial effects. Separately, GA as a natural reactive-oxygen-species-scavenger promotes antioxidation, and further enables excellent antibacterial effects and wet tissue adhesion due to a Schiff base and Michael addition reaction for accelerating wound healing. Once adhered to the wound, the precursor solution becomes both a physically and covalently cross-linked network hydrogel, which has potential advantages for wound healing with ease of use, external environment-isolating, and minimal tissue damage. The therapeutic effects of CS-GA-S on wound healing are demonstrated with the full thickness cutaneous wounds of a mouse model. The significant improvement of wound healing is achieved for mice treated with CS-GA-S. This preparation reduces wound system exposure, prolongs local drug residence time, and improves efficacy. Accordingly, with the incorporation of scolopin2 into the shape-adaptive CS-GA hydrogel, the composite hydrogel possesses multi-functions of mechanical adhesion, drug therapy, and skin wound healing. Overall, such an injectable or sprayable hydrogel plays an effective role in emergency wound treatment with the advantage of convenience and portability.

## 1. Introduction

The process of wound healing is largely hampered by excessive oxidative stress (ROS) in the injured wound [1]. ROS accumulated in the wound can not only induce strong inflammatory reactions and make the wound vulnerable, but also inhibit the function of endogenous stem cells and macrophages and hinder wound tissue regeneration [2]. In addition, ROS can significantly limit angiogenesis and lead to endothelial dysfunction [3,4]. Bacterial infection can cause significant damage to blood vessels and endothelial cells, leading to chronic wound formation. In addition, bacterial infections often lead to inadequate wound oxygenation and reduced nutrition, thereby accelerating and exacerbating the pathology of various wounds. Healthy ROS levels and a bacteria-free environment are necessary to effectively heal a wound and prevent the formation of ulcers. Therefore, the research and development of functional materials that remove ROS, with anti-inflammatory and bacteriostatic qualities, to effectively regulate the wound microenvironment and provide a microenvironment conducive to wound healing, is currently a hot topic in wound management.

The general principle of wound treatment is to transform the local pathological state of wounds into a physiological state and create a microenvironment suitable for wound repair [5,6]. The existing topical formulations for wounds are generally wipes, clothing contaminations, inaccurate dosages, etc. In addition, they generally smell pungent, which makes patients have a bad odor experience and affects the medication compliance of patients. Traditional hydrogel dressings on the market do not meet the special requirements of hydrogels for dynamic wounds. It is necessary to develop a kind of physical and chemical double cross-linking multifunctional hydrogel with shape adaptability, self-healing properties, and strong adhesion. Through molecular and chemical structure design, the sprayable self-healing hydrogels can possess good tissue adhesion, mechanical properties, and biological efficacy. Therefore, the design of a self-healing hydrogel based on the wound microenvironment with an adjustable size to adapt to the size change of a wound plays a crucial role in wound repair.

The shape adaptability and adhesion are very important for local regulation, but how to ensure that the drug stays in the local microenvironment for a long time and can fully protect the wound is the main issue. The key to improve the efficacy is to make the drug stay for a long time, and the shape of the preparation can be adjusted to achieve the expected efficacy.

Hydrogels are a class of highly hydrophilic biomaterials with complex network structures that are widely used as drug carriers for various diseases. They make contact with the wound and form a hydrogel to enable debridement and wound moisture. The application of traditional materials is often limited by various factors, such as having low efficiency, refractory, toxic, and non-waterproof qualities. Therefore, the research and development of environmentally friendly, high-performance adhesive hydrogels has become the urgent task in related application fields. Dopamine secreted by mussels have excellent biological properties such as strong adhesion, biocompatibility, degradability, and self-repair, which can be applied in many fields such as medicine and material science. Based on the characteristics of the chemical structure of dopamine, a natural plant compound—gallic acid, a kind of the biomimetic underwater adhesion material—has robust underwater adhesion characteristics similar to mussels. The pyrogallol group of gallic acid can realize rapid cross-linking of gallic acid (GA)-based polymers by means of oxidation and pH control to form biocompatible hydrogels with the following advantages: (i) many members of the pyrogallol group have a variety of bonding functional structures, which are cross-linked by hydrogen ion bonding or hydrophobic interaction [7,8]; (ii) they can covalently interact with amino groups to improve the mechanical properties of polymer material adhesives [8,9]; (iii) pyrogallol can chemically bond with tissues to produce strong tissue adhesives during oxidation [9]; (iv) innate antimicrobial capacity [10]; and (v) antioxidant capacity [11]. 

Peptides from the body and venom of centipedes, such as scolopendrisin 1, scolopin 1 and scolopin 2, have been reported [12]. The novel antimicrobial peptide scolopendin 2 was recently identified in the centipede body, and it exhibited broad antimicrobial activity by inducing membrane disruption [13]. In the present study, an intracellular stress response to scolopendin 2 from *Scolopendra subspinipes mutilans* was investigated in *Candida albicans*. The present data demonstrated glutathione depletion, ROS generation, and lipid peroxidation following treatment with scolopendin 2, which indicated that the peptide induced oxidative stress [14]. Reducing excessive oxidative stress in the wound and inhibiting bacterial growth are very important for wound healing [15]. Therefore, we chose scolopin2 as the model drug in our study.

**Figure 1:**
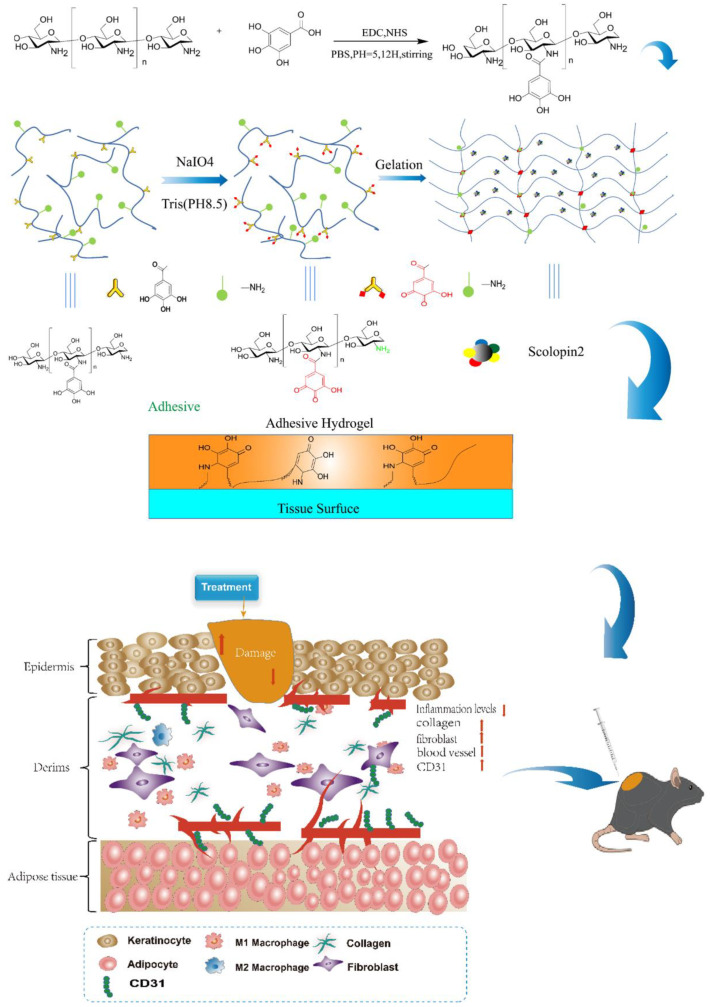
Schematic representation of shape-adaptive adhesive hydrogel loaded with scolopin2 for wound repair.

Herein, we plan to develop a hydrogel cross-linked by sodium periodate linker based on natural chitosan (CS) and GA (Figure 1). The hydrogel has multifunctional properties, including self-healing, tissue adhesion, and strong antimicrobial properties. At the same time, hydrogels can be formed in situ with shape regulation. In addition, adhesive hydrogels can accelerate wound repair by mechanical contraction. Stress remodeling around the wound can provide a better mechanical environment to support wound recovery, which can accelerate the formation of epithelial tissue, gradually reduce the wound surface, and release tension. We evaluated in detail the gelation behaviors, micro-morphology, degradation profile, mechanical properties, tissue adhesive, and self-healing capabilities of the system. The in vitro cell studies were employed to evaluate the cell compatibility, antibacterial, and anti-oxidative effect. Furthermore, through an acute mechanical wound model on mice, the promotion of the skin tissue healing was carefully studied. Overall, adequate mechanical strength, favorable tissue adhesiveness and self-healing ability, excellent antibacterial and antioxidation effects, and efficient therapeutic activity, as well as a facile dressing change, are integrated into one system for the application of chronic wound healing.

## 2. Results

### 2.1. Characterization of Chitosan-Gallic Acid

#### 2.1.1. Characterization

**Figure 2:**
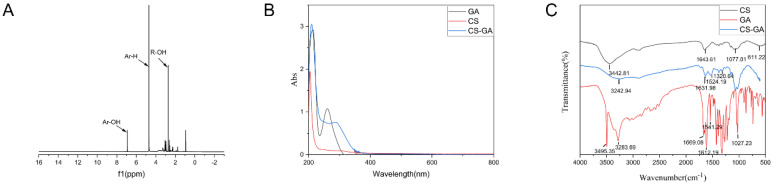
Characterization of CS-GA. (**A**) ^1^H-NMR spectrum of CS-GA; (**B**) UV−vis spectra of CS-GA, GA, and CS; (**C**) FT-IR spectrum of CS, GA, and CS-GA.

The chemical structure of chitosan-gallic acid (CS-GA) was confirmed by hydrogen nuclear magnetic resonance (^1^H NMR), Fourier transform infrared spectroscopy (FT-IR) spectra, and ultraviolet–visible spectrum (UV-vis) analysis (Figure 2A–C). Research shows that GA only exhibited a single peak at 7.1 ppm, which corresponded to the phenyl protons on the benzene ring [16]. In the ^1^H NMR spectrum of CS, two characteristic peaks at 4.9 and 4.7 ppm were assigned to the H of the D-glucosamine unit and the N-acetyl protons of the N-acetyl-D-glucosamine unit, respectively. The multiple peaks at 3.4–4.0 ppm corresponded to the skeleton hydrogen of CS [17]. Compared to CS, the ^1^H NMR spectra of the CS-GA showed that 4.71ppm is the characteristic peak of benzene ring hydrogen, and 6.92ppm is the characteristic peak of phenol hydrogen. [18], which was assigned to the phenyl protons of GA (Figure 1A). In the FT-IR spectrum as shown in Figure 1C, GA showed characteristic bands at 3495.35 and 3283.69 cm^−1^ (O-H stretching on the benzene ring), 1669.08 cm^−1^ (C=O stretching of carboxyl groups), 1612.19 and 1541.29 cm^−1^ (C=C stretching of the aromatic ring), 1200–1300 cm^−1^ (C-O/C-C stretching), and 1027.23 cm^−1^ (C-H deformation of the aromatic ring [16]. The FT-IR spectrum of CS-GA presented the characteristic absorption bands at 3243.94 cm^−1^, 1631.98 cm^−1^, 1524.19 cm^−1^, and 1320.64 cm^−1^ assigned to amide groups [19]; the absorption bands at 1631.98 cm^−1^, 1524.19 cm^−1^, and 1320.64 cm^−1^ were amide I, amide II, and amide III, respectively [20]. Whereas, the FT-IR spectrum of chitosan only presented the characteristic absorption bands of the amino group at 3442.81 cm^−1^, 1643.61 cm^−1^, 1077.01 cm^−1^, and 612.22 cm^−1^ [21]. The results showed that the amino group of chitosan was successfully combined with the carboxyl group of GA to form an amide group. The UV−vis of GA showed a characteristic absorption peak of catechol group at 260 nm. The UV−vis of CS modified by GA also showed a characteristic absorption peak of the catechol group at 280 nm (Figure 1B) [22]. The band occurred at a redshift. The catechol group content of CS-GA was 6.76 ± 0.03%. All of the above results confirmed that the CS-GA was successfully synthesized.

#### 2.1.2. Antioxidant Performance of CS-GA

**Figure 3:**
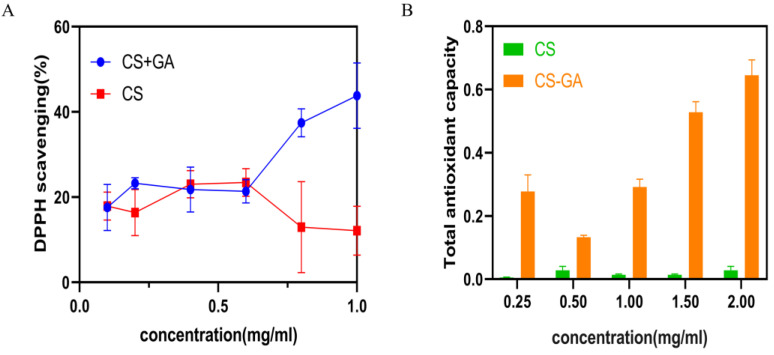
Antioxidant performance of the CS-GA. (**A**) The DPPH scavenging efficiency of CS and CS-GA; (**B**) the total antioxidant capacity of CS and CS-GA.

The antioxidant activity was evaluated by testing the efficiency of materials to scavenge the stable free radical 1,1-diphenyl-2-picrylhydrazyl (DPPH) and the total antioxidant capacity. The results were shown in Figure 3A,B. As shown in Figure 3A, all the groups of CS and CS-GA showed a similar degree of free radical scavenging efficiency at low concentration (0.1~0.6 mg/mL). The free radical scavenging efficiency of hydrogel material is significantly better than that of CS above 0.6 mg/mL. As the CS-GA concentration increased from 0.6 to 1 mg/mL, the DPPH scavenging rate efficiency gradually increased from 20.7% to 40%, which was significantly better than that of CS. As shown in Figure 3B, the total antioxidant capacity of all the groups of CS-GA was significantly better than that of CS. All of the above results confirmed that the CS-GA possessed excellent antioxidant activity.

### 2.2. Characterization of Hydrogel

#### 2.2.1. The Shape-Adaptive Performance of Hydrogel

**Figure 4:**
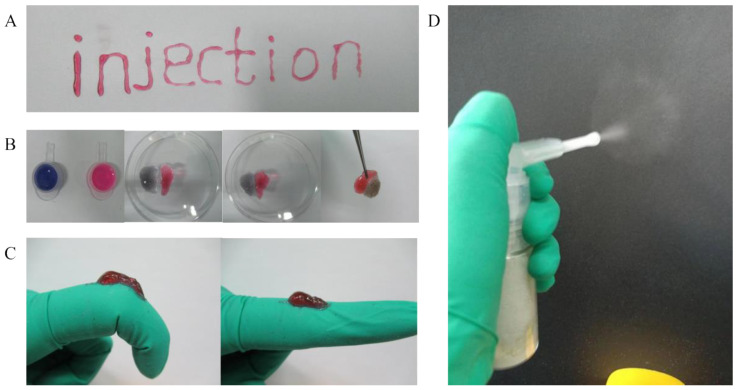
(**A**) The injectability, (**B**) self-healing, Red gel is the gel stained with Rhodamine B and blue gel is the gel stained with methyl blue (**C**) shape adaptation, and (**D**) spray ability of hydrogel.

Figure 4A and Supplementary Video S1 showed the word “injection” which was written by pushing the hydrogel solution from the syringe, and the hydrogel still maintained good hydrogel morphology without any clogging and dissolution. In Figure 4B, two pieces of hydrogel were put together and placed for 30 min at room temperature. The hydrogel automatically healed into a complete hydrogel. As shown in Figure 4C, the hydrogel could be deformed with the bending and extension of the finger joint without breaking. As shown in Figure 4D and the Supplementary Video S2, the hydrogel lyophilized powder particles can be sprayed evenly in the shape of an “umbrella”.

#### 2.2.2. Scanning Electron Microscopy

**Figure 5:**
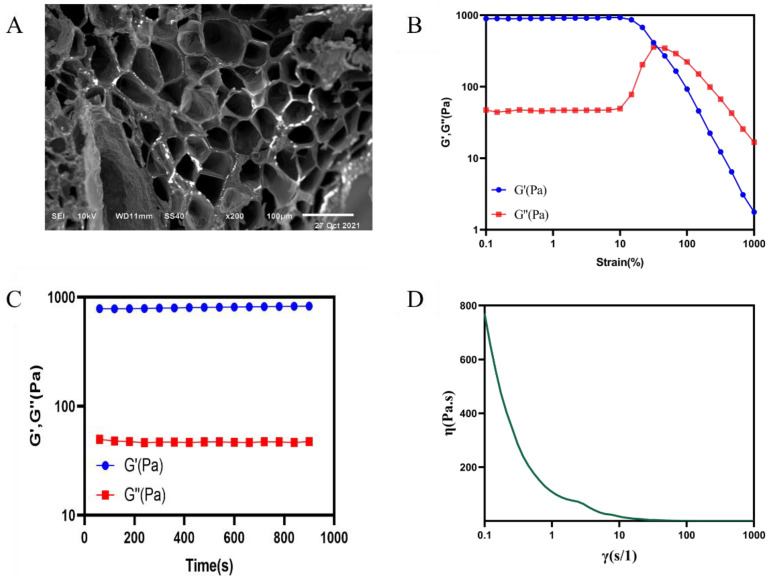
SEM and rheological properties of hydrogel. (**A**) SEM morphology of hydrogel. Rheological measurements of the hydrogel. (**B**) Strain amplitude sweep test of hydrogel with a constant frequency of 10 rads^−1^ and a varying of the strain from 0.1% to 1000%. (**C**) Dynamic time sweep of the hydrogel, at a strain of 1%. (**D**) Viscosity curve of hydrogel. The concentration of the hydrogel was 40 mg mL^−1^ (17.6 mM), T = 25 °C.

The hydrogel was characterized by a scanning electron microscope (SEM) as shown in Figure 5A. The hydrogel presented a regular porous three-dimensional reticular structure.

#### 2.2.3. Rheological Properties of the Hydrogel

Rheological properties of the hydrogel were shown in Figure 5. Figure 5B showed the strain amplitude sweep test result. Storage modulus (G′) values of the hydrogel remained unchanged and larger than the loss modulus (G″) at first, while the G′ curve intersected with the G″ curve at about 50% of the strain, indicating the critical point near which the hydrogel was between the states of solid and fluid. Then, the G′ of hydrogel dramatically decreased and went lower than G″, which showed that the hydrogel network collapsed [23]. When the strain was retracted to 1%, both the G′ and G″ of the hydrogel quickly returned to the initial state (Figure 5C). Viscosity curve of the hydrogel showed that the hydrogel possessed shear thinning property (Figure 5D).

#### 2.2.4. Degradation Rate and Swelling Ratio of Hydrogel

**Figure 6:**
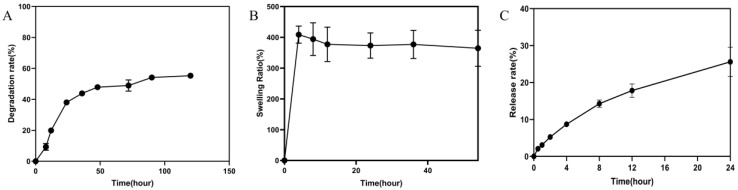
Degradation rate and swelling ratio of the hydrogel. (**A**) The degradation rate and (**B**) swelling ratio of the hydrogel. (**C**) The release rate of the scolopin2-loaded hydrogel.

The degradation rate and swelling ratio of the hydrogel were shown in Figure 6A,B. The hydrogel degraded rapidly in 0~24 h. The degradation rate reached 38%. The degradation rate slowed down in 25~48 h. The degradation rate tended to be at a flat level in 48~120 h. The freeze-dried hydrogel reached swelling equilibrium after 2 h, and the swelling rate was about 300%.

#### 2.2.5. In Vitro Drug Release Study

The results were shown in Figure 6C. We can see that the process of drug release from the gel is a slow-release process through the release assay within 24 h. The release rate could reach about 25% at the 24 h.

#### 2.2.6. Antimicrobial Assay

**Figure 7:**
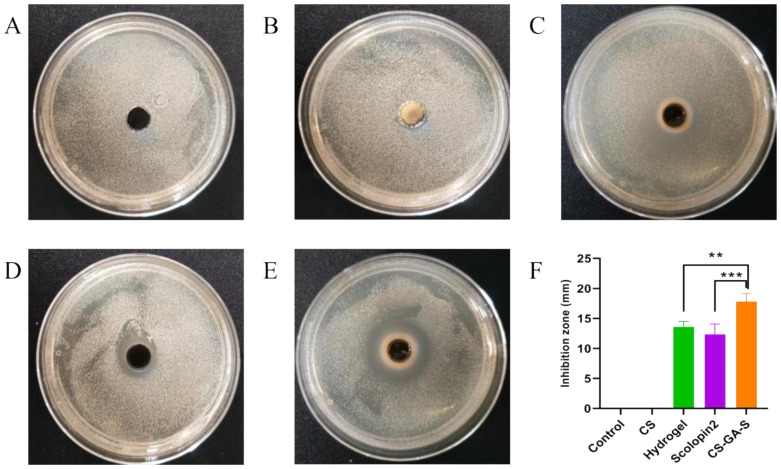
The antimicrobial efficacy of the hydrogel. (**A**) Model group, (**B**) CS group, (**C**) hydrogel group, (**D**) scolopin2 group, (**E**) multifunctional hydrogel (CS-GA-S) group, and (**F**) the size inhibition zone of all groups. ** represents *p* < 0.005, *** represents *p* < 0.0005.

The results were shown in Figure 7. CS did not show obvious antibacterial activity under the experimental conditions, but the hydrogel, multifunctional hydrogel (CS-GA-S), and scolopin2 all showed obvious antibacterial effects. The size of the inhibition zone of the hydrogel and CS-GA-S was 13.61 ± 0.88 mm and 17.82 ± 1.31 mm, respectively. The size of scolopin2 was 12.33 ± 1.74 mm. The antibacterial effect of CS-GA-S was significantly better than that of the hydrogel, and the antibacterial effect of CS-GA-S was also better than that of scolopin2 alone.

#### 2.2.7. Cytocompatibility of Hydrogel

**Figure 8:**
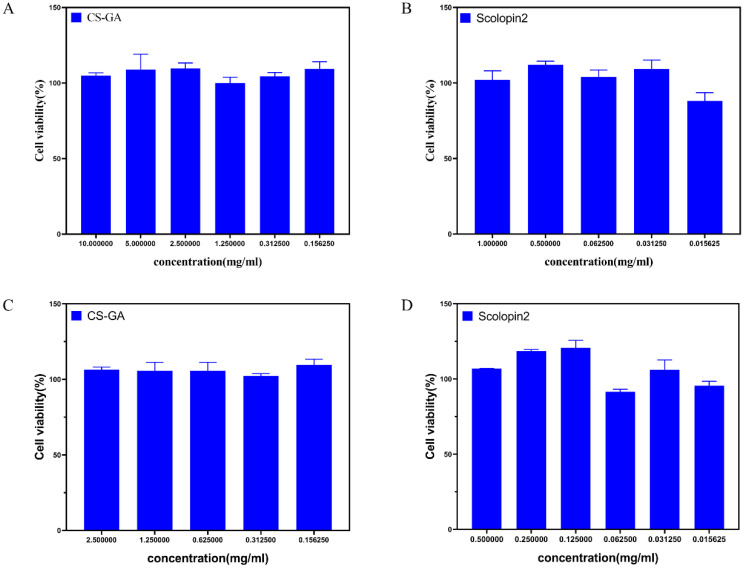
The NIH3T3 cell viability with (**A**) CS-GA and (**B**) scolopin2. The Caco-2 cell viability with (**C**) CS-GA and (**D**) scolopin2.

As shown in Figure 8, the hydrogel and scolopin2 showed good cell viability (more than 100%) when varying the concentrations of the hydrogel from 10 mg/mL to 15.625 µg/mL (Figure 8A) and the concentrations of scolopin2 from 1 mg/mL to 15.625 µg/mL (Figure 8A). This indicated that the hydrogel and scolopin2 had no obvious cytotoxicity. The hydrogel and scolopin2 showed good Caco-2 cell viability (more than 80%) when varying the concentrations of the hydrogel from 2.5 mg/mL to 15.625 µg/mL (Figure 8C) and the concentrations of scolopin2 from 0.5 mg/mL to 15.625 µg/mL (Figure 8D). This indicated that the hydrogel and scolopin2 had no obvious cytotoxicity.

### 2.3. In Vivo Assessment of the Wound Healing Activity

#### 2.3.1. Effect of Hydrogel on Wound Closure in Mice

**Figure 9:**
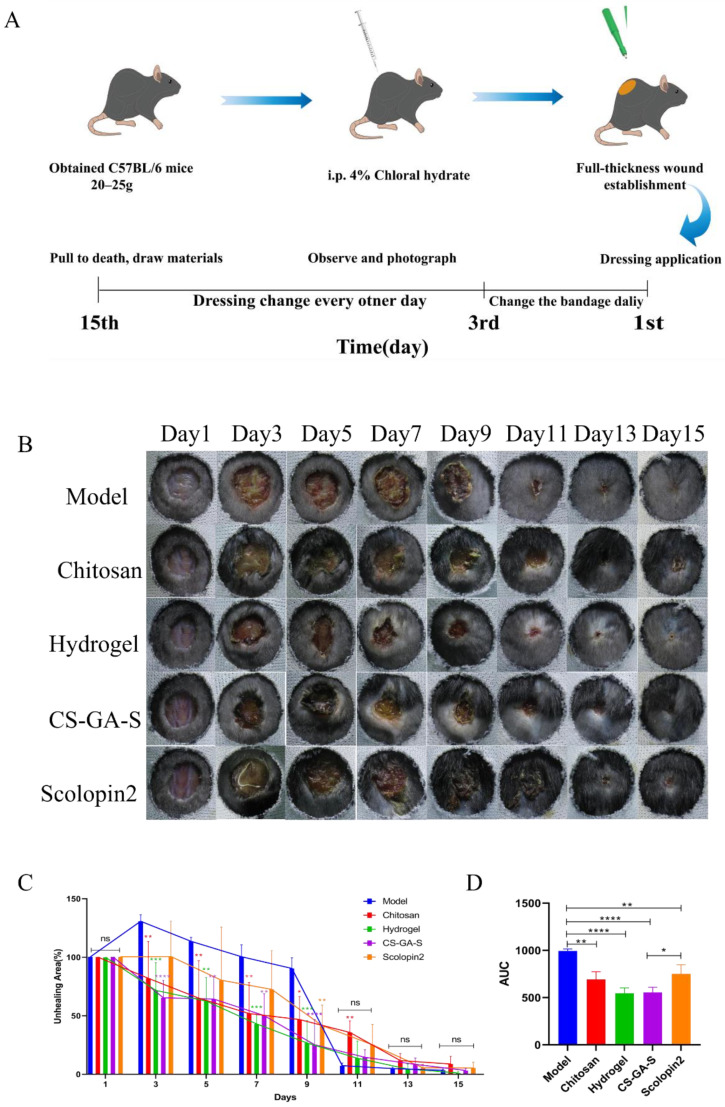
In vivo wound healing evaluation with wound mice. (**A**) Model building and drug administration process. (**B**) The wound area representative photos of the mice of all the five groups in the time of treatment. (**C**) The bar chart and line chart of the wound area. (**D**) The quantified area under the curve (AUC). ns represents no significance, * represents *p* < 0.05, ** represents *p* < 0.005, *** represents *p* < 0.0005, **** represents *p* < 0.0001.

The method of the animal model establishment and drug administration is shown in Figure 9A. The wound area representative photos of the mice of all the five groups on days 1, 3, 5, 7, 9, 11, 13, and 15 are given in Figure 9B. Furthermore, the quantitative wound area in the experimental time frame was measured based on the wound photos. The unhealing percentage of the wound of all groups was found to decrease in a time-dependent fashion (Figure 9C). Wound healing was markedly faster in both hydrogel and CS-GA-S groups as compared to the model groups as they had a highly significant greater area under the curve (AUC) throughout the study period, and the effect of the CS-GA-S group was better than scolopin2 alone (Figure 9D). As shown in Figure 9C, compared to the model group, chitosan, the hydrogel, and CS-GA-S significantly reduced the wound area in the first seven days. Especially regarding the hydrogel, on the third day, the wound areas in the model group remained as high as 130.5%, but 71.7% and 65.1% of the contraction in the wound area was achieved with the hydrogel and CS-GA-S dressing, which was also smaller than that of the chitosan dressing group (81.9%). On the 9th day, the wound area of all medication groups was smaller than the model group. Additionally, the CS-GA-S group had highly significant differences from the model group, followed by the hydrogel group, scolopin2 group, and, last, the CS group.

#### 2.3.2. Histological Analysis

**Figure 10:**
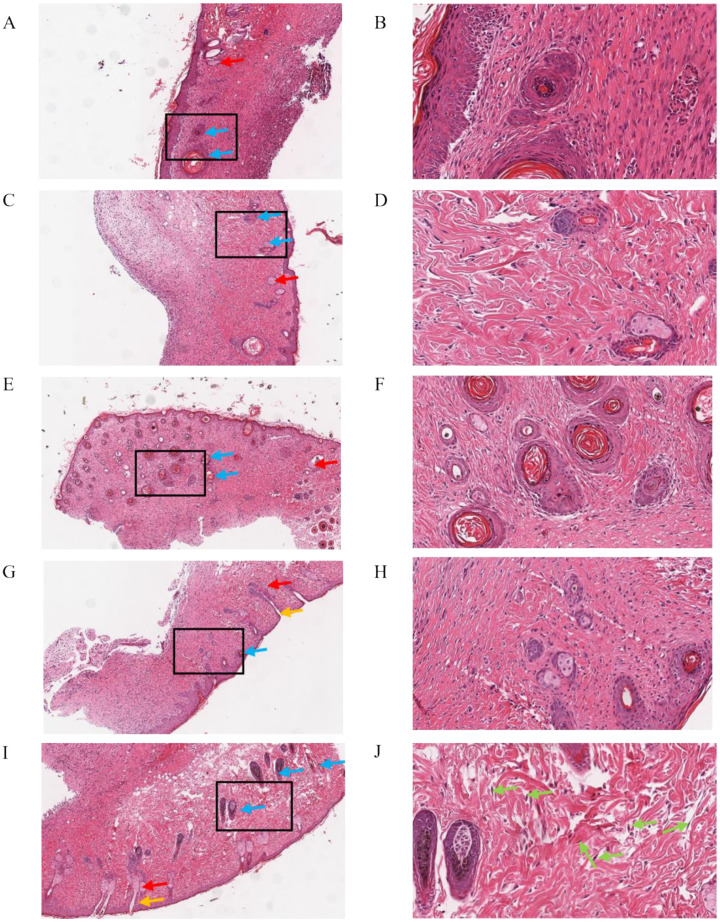
Histopathological examination of the healed tissue. Microscopic images of skin, hematoxylin and eosin (H&E)-stained (**A**) (×200) and (**B**) (×400) model group; (**C**) (×200) and (**D**) (×400) CS group; (**E**) (×200) and (**F**) (×400) hydrogel group; (**G**) (×200) and (**H**) (×400) scolopin2 group; (**I**) (×200); and (**J**) (×400) the multifunctional hydrogel (CS-GA-S) group. Blue arrows represent blood vessels, red arrows represent sebaceous glands, orange arrows represent hair follicles, and green arrows represent fibroblasts.

**Figure 11:**
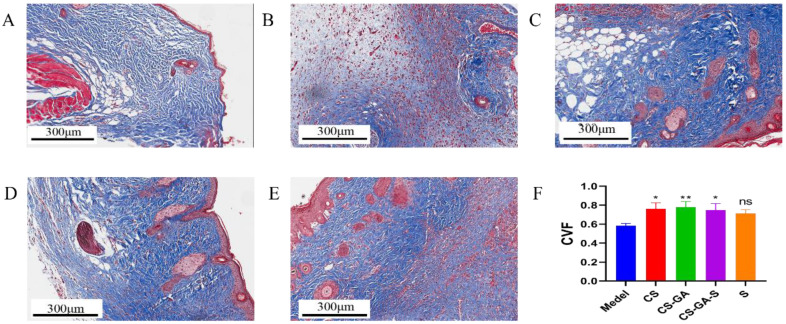
Masson staining microscopic images of skin. (**A**) Model group, (**B**) CS group, (**C**) hydrogel group, (**D**) scolopin2 group, and (**E**) CS-GA-S group. (**F**) The collagen volume fraction (CVF). Scale bar: 300 μm. ns represents no significance, * represents *p* < 0.05, ** represents *p* < 0.005.

To evaluate the healing effect of the dressings from a histological perspective, hematoxylin and eosin (H&E) and Masson trichrome staining were applied on regenerated skins collected. The result of H&E is shown in Figure 10. The model group showed a certain degree of re-epithelialization, inflammatory cell infiltration, and showed a few fibroblasts’ proliferation and angiogenesis. Relatively, the dermis was thin. The CS group and scolopin2 group showed complete re-epithelialization and a thinner dermis, but showed a high degree of inflammatory cell infiltration and a lower degree of fibroblast proliferation and a small amount of angiogenesis. The hydrogel group showed inflammatory cell infiltration, but formed intact dermal tissue, completed re-epithelialization, and showed more angiogenesis and proliferation. The CS-GA-S showed great healing efficacy, and the complete regeneration of the skin appendages and dermal tissue were observed, presenting the largest number of hair follicles and the greatest amount of fibroblasts going through proliferation and angiogenesis. Meanwhile, no obvious inflammatory cell infiltration was observed. Figure 11 showed the Masson trichrome staining results: the arrangement of collagen in the model group was disordered. The group of CS, the hydrogel, and CS-GA-S had a higher number of collagen depositions compared with the model group by calculating the collagen volume fraction (CVF) (Figure 11F).

#### 2.3.3. Immunohistochemistry

**Figure 12:**
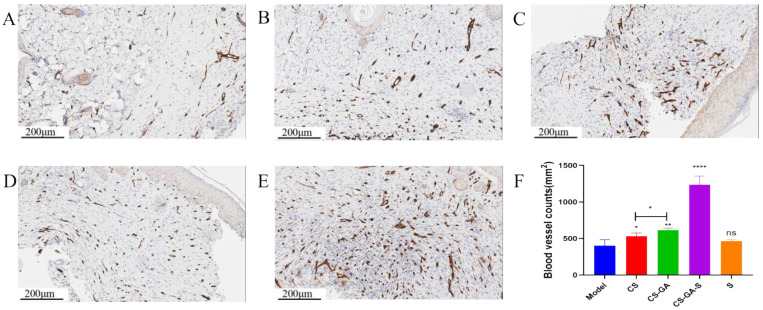
Representative IHC images of regenerated wound tissues with anti-CD31. (**A**) Model group, (**B**) CS group, (**C**) hydrogel group, (**D**) scolopin2 group, and (**E**) CS-GA-S group. (**F**) The blood vessel counts. Scale bar: 200 μm. ns represents no significance, * represents *p* < 0.05, ** represents *p* < 0.005, **** represents *p* < 0.0001.

The immunohistochemical (IHC) staining results of CD31 are displayed in Figure 12. Compared to the model group, the CS-GA-S presented a strong positive staining signal of CD31, followed by the hydrogel group, CS group, and scolopin2 group. Importantly, the hydrogel group presented stronger positive staining of CD31 than the CS group.

**Figure 13:**
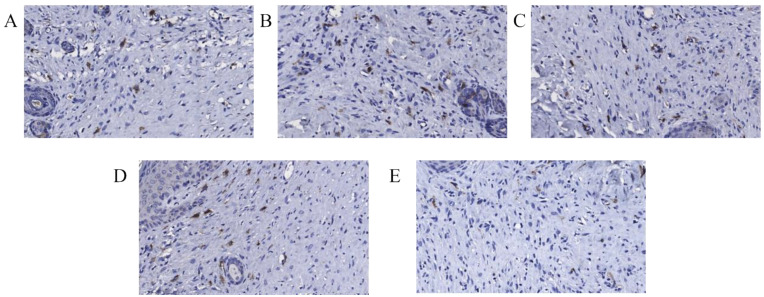
Representative IHC images of regenerated wound tissues with anti-c-kit. (**A**) Model group, (**B**) CS group, (**C**) hydrogel group, (**D**) scolopin2 group, and (**E**) CS-GA-S group (×1000).

The IHC staining results of the c-kit were displayed in Figure 13. There was a certain number of positive signal expression of mast cell in the model group, CS group, and CS-GA group, whereas almost no expression in CS-GA-S group.

**Figure 14:**
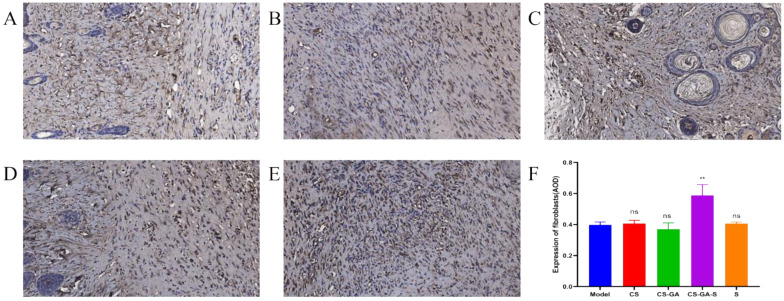
Representative IHC images of regenerated wound tissues with anti-HSP47. (**A**) Model group, (**B**) CS group, (**C**) hydrogel group, (**D**) scolopin2 group, and (**E**) CS-GA-S group (×600). (**F**) The expression of fibrocytes was semi-quantified. ns represents no significance, ** represents *p* < 0.005.

The IHC staining results of HSP47 were displayed in Figure 14. Compared to the model group, the CS-GA-S group presented a strong positive staining signal of HSP47, indicating that the CS-GA-S group has more fibroblasts, while the other groups are not significantly different from the model group.

**Figure 15:**
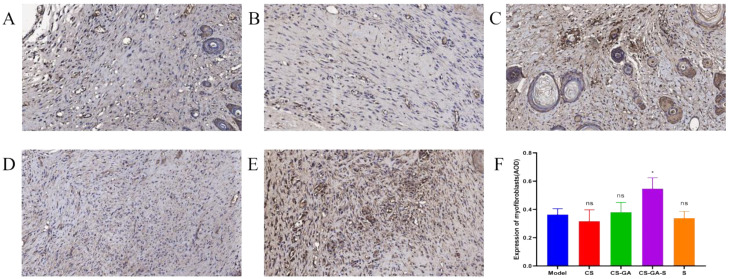
Representative IHC images of regenerated wound tissues with anti-α-SMA. (**A**) Model group, (**B**) CS group, (**C**) hydrogel group, (**D**) scolopin2 group, and (**E**) CS-GA-S group (×600). (**F**) The expression of myofibroblasts was semi-quantified. ns represents no significance, * represents *p* < 0.05.

The IHC staining results of α-SMA were displayed in Figure 15. Compared to the model group, the CS-GA-S group presented a strong positive staining signal of α-smooth muscle actin (α-SMA), indicating that the CS-GA-S group has more myofibroblasts, while the other groups are not significantly different from the model group.

## 3. Discussion

In this study, we synthesized a hydrogel material via modifying chitosan with polyphenol compound-GA. Sodium periodate was used to oxidize the catechol group connected to chitosan to benzoquinone group, and the benzoquinone group can combine with amino group through a Michael-type addition reaction to form a hydrogel network structure. Meanwhile, benzoquinone group can also interact with amino groups in tissue to produce tissue adhesion of the hydrogel [24]. The chemical structure of CS-GA was confirmed by ^1^H NMR, FT-IR spectra, and UV–vis spectra. Interestingly, we found the CS-GA’s solubility was splendid when catechol group contents in CS-GA were about 10%, but CS-GA’s solubility was reduced when catechol group contents in CS-GA were about 6%. We speculated that CS-GA’s solubility increases with the increase of catechol group contents in CS-GA within a certain range. 

The antioxidant performance results demonstrated that the hydrogel had excellent antioxidant activity. CS-GA has excellent antioxidant activity for catechol groups provided by GA [25]. Antioxidant wound dressings can improve the wound healing process by regulating the over-production of ROS [26]. Thus, the CS-GA could be used as an excellent antioxidant dressing to promote the skin wound repair. 

The self-healing capacity is important for hydrogel dressings to promote the skin wound repair because the dressing may bear external mechanical force after application onto the wound site. The results of rheological properties showed the hydrogel network was able to recover after it was collapsed when adjusting the strain from 1000% to 1%. The results prompted that the hydrogel had rapid and highly efficient self-healing capacity. Moreover, self-healing ability of the hydrogel was further demonstrated by the macroscopic self-healing ability (Figure 4B) [19]. The viscosity curve of hydrogel indicated that the hydrogel possessed a shear thinning property [26], which was beneficial for the injection of the hydrogel. In addition, a macroscopic injection experiment also demonstrated the injectability of hydrogel. The magnificent self-adapting feature provided more evidence for them being an excellent dressing for in vivo wound treatment, due to the shear thinning property and being automatically shape-adjustable without external stimuli to match the wound area [24]. At the same time, it is suggested that the hydrogel can be used not only in wound treatment, but also in joint and orthopedic diseases treatment. Interestingly, the hydrogel of the invention has the potential to be prepared as a sprayable hydrogel through the ejection experiment. It provided the possibility for the wide application of hydrogel. The hydrogel has a volume non-expanding property because the wet weights of the hydrogel were lower than the initial wet weights during the whole degradation process. The volume non-expanding property of the hydrogel could reduce physical compression on the wound site during application. The swelling ratio of hydrogel dressing results demonstrated that the hydrogel can retain a large amount of water to maintain a moist environment of the wound site. At the same time, the 3D network structure and high water content of the hydrogel are similar to the stromal and tissue structure of biological cells [27,28]. This is the advantage for wound dressing. Meanwhile, the prepared hydrogel had excellent antioxidant activity, which was beneficial for the wound repair.

The process of the wound healing contains inflammation, cell migration and proliferation, extracellular matrix deposition, and remodeling [29]. In this study, the promoting effect of hydrogel on wound healing was evaluated by establishing a model of acute mechanical trauma. Wound healing was markedly faster for both treatment groups than the model groups in the first nine days. More importantly, the treatment groups had obviously newborn skin tissue. However, the model group did not have any newborn skin tissue instead of scabs. Though the degree of wound healing of the treatment groups had no significant difference from the model group due to the model group’s scab peeling off in the next few days, the wound opened in the process of pulling the tail, just like the wound on the first day of modeling (Supplementary Figure S1), which speculated that pseudo healing occurred in the model group. The results showed that our hydrogel can promote wound healing.

It is now well-known that inflammation level is a crucial evaluation indicator during the process of wound healing and tissue regeneration. The inflammation phase in the chronic wound was severely prolonged or even unable to transform to the proliferation phase. The results of H&E staining showed that the hydrogel reduced the inflammatory response. This result was supported by a decrease in mast cells (MCs). MCs are granulated, immune cells of the myeloid lineage. MCs mediate various inflammatory responses due to the nature of their secretory products. They are involved in important physiological and pathophysiological responses related to inflammation, chronic wounds, and autoimmune diseases [30]. It is reported that mast cells can be proposed for a dual role in injury response. They release different types of mediators that stimulate angiogenesis, which induces the appearance of myofibroblasts promoting wound healing in the early stage of injury. However, hyperactivation of mast cells can induce scar formation and aggravate inflammatory reactions in the remodeling phase [31]. The staining results of mast cells demonstrated that the CS-GA-S reduces mast cell expression to a greater extent. Fibroblasts played an important role in wound healing. Fibroblasts were responsible for producing most of the collagen fibers to repair damaged skin, and the increase of fibroblasts contributes to the formation of hair follicles during wound healing and reduces the probability of scarring after skin healing [32,33]. HSP47 is a useful marker for skin fibroblasts in routine, paraffin specimens [34]. The results demonstrated that the CS-GA-S group had a high expression of HSP47 through the results of immunohistochemistry. Activated fibroblasts, also known as myofibroblasts (MF), contribute to wound healing by regulating the secretion of extracellular matrix (ECM), matrix metalloproteinases, tissue inhibitors of metalloproteinases, growth factors, and cytokines important for wound healing [35]. MF generate increased contractile force compared to fibroblasts [36]. Neo expression of α-SMA is an established marker for MF differentiation [37,38]. The results demonstrated that the CS-GA-S group had a high expression of α-SMA through the immunohistochemistry analysis.

As an attachment and support for cell growth, collagen can induce the proliferation, differentiation, and migration of epithelial cells [39]. Masson trichrome staining showed that there was more collagen deposition in the hydrogel groups, and the collagen was arranged orderly compared with the model group. This is beneficial for wound repair.

Blood vessels can provide nutrition to the cells associated with healing and sustain the growth of the newly-formed granulation tissue, and thus, the angiogenesis process is also vital for wound repair. CD31 is a kind of transmembrane protein expressed in early angiogenesis [40]. The results demonstrated that the hydrogel groups had a high expression of CD31 through the results of immunohistochemistry.

Additionally, during the process of wound healing, preventing the wound from being infected by bacteria can prevent the wound from further infection and deterioration, which results in poor healing. The results of antimicrobial assay showed that the hydrogel could inhibit the reproduction of bacteria and enhance the bacteriostatic ability of scolopin2.

The above results suggested that the application of the hydrogel dressing had a favorable influence on the various phases of diabetic wound healing and thus facilitated the healing process.

## 4. Materials and Methods

### 4.1. Materials

Chitosan, 1-(3-Dimethylaminopropyl)-3-ethylcarbodiimide hydrochloride (EDC), N-hydroxy succinimide (NHS), and GA were purchased from Sigma-Aldrich Chemie (Darmstadt, Germany). Organic solvents, including absolute ethanol, and inorganic solvents, including hydrochloric acid and sodium hydroxide, were provided at reagent grade, and used without purification. Phosphate buffer (1X) was purchased from Solarbio Science and Technology (Beijing, China). Tris was purchased from Biotopped (Beijing, China). Scolopin2 was purchased from Nanjing Pepide Biotech (Nanjing, China). The peptide sequence is GILKKMLHRGTKVYMRTLSKRSH. Sodium periodate was purchased from Fuchen (Tianjin, China).

### 4.2. Synthesis of GA

After dissolving 250 mg chitosan in HCl (2.5 mL 5 M) and stirring more than 4 h, and adding 20 mL of phosphate buffer (PBS) and stirring overnight, 2.5 mL sodium hydroxide solution (NaOH, 5 M) was added to adjust the solution pH to 5. Amounts of 622 mg EDC and 374 mg N-hydroxy succinimide (NHS), and 23.6 mg/mL of gallic acid solution with a 12.5 mL (anhydrous ethanol: PBS buffer = 1:1) concentration, were added to the above solution. Then, the reaction solution pH was adjusted to 5 and was stirred for 12 h. Dialysis was performed after the reaction. The dialysate was a PBS buffer with a pH of 5. The dialysate was changed every 3 h. After about 5 times, the dialysate was dialyzed with distilled water with a pH of 5. Finally, the reaction solution completed by dialysis was dried under lyophilization for 48 h using freezer dryer (CHRIST, Alpha 2-4LSCplus, Osterode, Germany) and CS-GA was obtained.

### 4.3. Characterization of GA

#### 4.3.1. Characterization

^1^H NMR (600 MHz) spectra of the CS-GA was tested by employing a Bruker Avance 600 MHz NMR instrument. D_2_O was used as the solvent and internal standard (4.70 ppm) of the CS-GA. The FT-IR spectra of CS-GA and CS were recorded in the range of 4000–400 cm^−1^ using a Nicolet IS10 FT-IR spectrometer. UV–visible spectra of CS-GA, CS, and GA in the range of 800–200 nm was recorded using an Agilent Cary 100 UV–Vis spectrophotometer. Meanwhile, the catechol group content in CS-GA was calculated by determining the absorbance of CS-GA at 280 nm using an Agilent Cary 100 UV–Vis spectrophotometer.

#### 4.3.2. Antioxidant Properties of the Chitosan-Gallic Acid

The antioxidant activity of the GA was evaluated by testing its capability to scavenge the stable DPPH free radicals [26]. A CS solution and a CS-GA solution with a series of concentrations (0.1 mg/mL, 0.2 mg/mL, 0.4 mg/mL, 0.6 mg/mL, 0.8 mg/mL, 1.0 mg/mL) were prepared, and 0.006 g DPPH powder was dissolved in 50 mL anhydrous ethanol and preserved away from light. The mixture was stirred in 96-well plates (Costar) and incubated in a dark place for 30 min. The absorbance was measured at 517 nm using a microplate reader. The degradation of DPPH was calculated via the following equation: DPPH scavenging % = (1 – (A_sample_ − A_blank_)/A_control_) × 100, where A_sample_, A_blank_, A_control_ are the absorption of samples (DPPH + ethanol + sample), the absorption of control (DPPH + ethanol + the dissolvent of sample), and the absorption of blank (DPPH + ethanol), respectively. The antioxidant activity of the CS-GA was also evaluated by calculating the total antioxidant capacity [41]. A CS solution and a CS-GA solution of a series of concentrations (0.25 mg/mL, 0.5 mg/mL, 1 mg/mL, 1.5 mg/mL, 2.0 mg/mL) were prepared. Then, the total antioxidant capacity using the total antioxidant capacity assay kit with FRAP method (Beyotime, Shanghai, China) was determined.

### 4.4. Preparation of Adhesive Hydrogel

For preparation of the hydrogel, CS-GA was dissolved in 0.2 M Tris, which made the pH 8.5. Sodium periodate was added to the solution and the blank hydrogel was placed in a static form [42]. The mass ratio of CS-GA to sodium periodate was 40:1. For preparation of the drug-loaded hydrogel, scolopin2 was first dissolved in 0.2 M Tris, and then CS-GA was added to the above solution. The remaining steps were the same as the preparation of the blank hydrogel. The concentration of scolopin2 was 10 mg per milliliter of hydrogel.

### 4.5. Characterization of Adhesive Hydrogel

#### 4.5.1. The Injectability of Hydrogel

The injectability of the hydrogel was evaluated by placing the precursor solution in a 1 mL syringe, and injecting it and writing the word “injection” after the solution formed hydrogel. The injection process was photographed and videotaped [43].

#### 4.5.2. The Self-Healing of Hydrogel

The two kinds of hydrogel were stained with rhodamine B and methyl blue, respectively. Half of them were cut off and placed in the same Petri dish to observe the self-healing phenomenon. Photographs of the self-healing process of the hydrogel were taken [42].

#### 4.5.3. The Shape Adaptation of Hydrogel

In order to elucidate the hydrogel’s shape adaptation, the hydrogel was placed at the knuckles, and then the knuckles were bent and stretched to observe the change of the hydrogel. The process was photographed.

#### 4.5.4. The Sprayability of Hydrogel

The lyophilized hydrogel was grounded into powder using a tissue homogenizer. The rotational speed of the homogenizer was set at 2000 rpm and recycled for 10 times. The obtained powder was passed through the sieve and screened out; it could pass through the 80-mesh sieve but not the 100-mesh sieve. The spray test was carried out using a long-nozzle powder spray bottle. The spray process was photographed and videotaped.

#### 4.5.5. Scanning Electron Microscopy (SEM)

The morphology of the freeze-dried hydrogel was observed by SEM (JSM6510LV, JEOL, Osaka, Japan). Before observation, the surface of the hydrogel was sprayed with a gold layer.

#### 4.5.6. Rheological Properties of Hydrogel

The rheological properties of the hydrogel were evaluated using an Anton Paar rotational rheometer (MCR92) via three different methods. An appropriate amount of sample was placed on the sample table, the type of the test rotor was a 50 mm parallel plate, the gap was 1 mm, and the test temperature was 25 °C. (1) The strain amplitude sweep test (γ = 0.1–1000%) was performed to detect the hydrogel’s critical strain point. (2) The test strain was adjusted back to 1% for a time-modulus scanning test. (3) A shear thinning test with the shear rate varying from 0.1 s^−1^ to 1000 s^−1^ was performed to study the effect of shear rate on the hydrogel’s viscosity.

#### 4.5.7. Degradation Rate of Hydrogel

The hydrogel was immersed into PBS (0.01 M pH 7.4) and then placed on a 37 °C shaker with a shaking speed of 100 rpm. When reaching the pre-set time point, the superficial water was removed using a filter paper and the hydrogel was weighed [29]. The pre-time was 0 h, 6 h, 12 h, 24 h, 36 h, 48 h, 72 h, 96 h, 120 h. Degradation rate was calculated using the following equation: Degradation rate (%) = (W_0_ − W_t_)/W_0_ × 100%, where W_0_ and W_t_ represent the initial weight of the hydrogel and the weight at the pre-set time point, respectively.

#### 4.5.8. Swelling Ratio of Hydrogel

The freeze-dried hydrogel was immersed into PBS (0.01 M pH 7.4) and then placed at 37 °C on a shaker with a shaking speed of 100 rpm [44]. When reaching the pre-set time point, the superficial water was removed using a filter paper and the hydrogel was weighed. The pre-time point was 0 h, 2 h, 8 h, 12 h, 24 h, 48 h, 54 h. Swelling ratio was calculated using the following equation: Swelling ratio (%) = (W_t_ − W_0_)/W_0_ × 100, where W_t_ and W_0_ represent the initial weight of the hydrogel and the weight after at the pre-set time point, respectively.

#### 4.5.9. In Vitro Drug Release Study

Scolopin2 was mixed with hydrogel precursor solution to form a stable hydrogel. Then, the hydrogel was placed in a dialysis bag and dipped in 10 mL of the release medium (phosphate buffer, PBS). Further, it was placed into a constant temperature culture shaker (THZ-103B, Shanghai, China) with a set temperature of 37 °C and a speed of 100 rpm. At the set time interval, 0.5 mL of release media was removed and another 0.5 mL of fresh release media was added. The release media measured the content of scolopin2 using high-performance liquid chromatography (Agilent 1260 Infinity, Palo Alto, CA, USA). The mobile phase was A (0.1% Trifluoroacetic acid (TFA) in Acetonitrile), and B (0.1% TFA in H_2_O); gradient elution was 20–45% A in 0–5 min, 45% A in 5–7 min; liquid velocity was 1 mL/min; and injection volume was 10 μL.

#### 4.5.10. Antibacterial Efficacy of Hydrogel

The radial diffusion assay was used to elucidate bacteriostatic ability of the hydrogel and scolopin2 [45]. *Staphylococcus aureus* (*S. aureus*) was grown to a mid-logarithmic degree, and then the bacteria suspension was diluted to 4 × 10^6^ CFU/mL. An amount of 100 μL diluted bacteria suspension was added into 6 cm Petri dishes, spread evenly and with holes punched in the middle of the plate. The plates were then prepared by punching 8 mm holes using a biopsy punch. Chitosan solution, blank hydrogel, CS-GA-S, and scolopin2 solution were then added to holes on the agarose and incubated at 37 °C for 12 h. Three plates were repeated for each drug. The diameter of antibacterial zone was calculated by taking pictures of the bacterial colony.

#### 4.5.11. Cytocompatibility of Hydrogel

The viability of Caco-2 cells and NIH3T3 cells subjected to the hydrogel and scolopin2 was measured via CCK8 assay (Solarbio) [46,47]. The complete growth medium was Dulbecco’s modified Eagle’s medium (DMEM) (Gibco, Grand Island, NY, USA ) supplemented with 10% fetal bovine serum (Gibco), 1.0 × 10^5^ U/L penicillin (Gibco), and 10 mg/mL streptomycin (Hyclone). Caco-2 cells or NIH3T3 cells were seeded in a 96-well plate (Costar) with a density of 10,000 cells per well and cultured in a humidified atmosphere containing 5% CO_2_ at 37 °C. After being cultured for 24 h, the culture medium was removed and a culture medium with different amounts of hydrogel and scolopin2 was added. After 12 h, the medium was removed and 100 μL of medium with 10 μL of CCK8 reagent was added into each well. After incubation for 2 h at 37 °C, its absorbance at 450 nm was determined using a microplate reader. The cell viability was calculated by the following equation: cell viability (%) = (A_s_ − A_0_)/(A_0-s_ − A_0_) ×100, where A_s_, A_0_, A_0-s_ represent the absorption of the test group (cell + CCK8 reagent + sample), the absorption of blank group (medium + CCK8 reagent), the absorption of control group (cell + CCK8 reagent), respectively.

### 4.6. In Vivo Assessment of Wound Healing Activity

#### 4.6.1. Animals

A total number of 20 male C57BL/6 rats (25–27 g) were utilized for the wound healing experimental study. Animals were kept for a 1-week acclimatization at the animal house. They had free access to drinking water and food. All protocols were performed upon approval by the Committee of Animal Experimental of the Peking Union Medical College.

#### 4.6.2. Excision Wound

The mice were anesthetized by an intraperitoneal injection of 4% chloral hydrate, and then the dorsa of the mice were shaved. One full thickness wound with a diameter of 8 mm, deep into the fascia, was made on the dorsal region of the mice by using an 8 mm biopsy punch.

#### 4.6.3. Experimental Design

Mice were randomly grouped into 5 groups (4 rats/group). One group served as the model group and the wound of these mice was left without treatment. The rest of the groups received chitosan (100 μL, 40 mg/mL), hydrogel (100 μL, 40 mg/mL), CS-GA-S, and scolopin2 (100 μL, 10 mg/mL), respectively. All groups were simply bandaged with aseptic gauze after treatment. All treatment was performed every day in the first three days. Then, all treatments were performed every other day. For wound area monitoring, on the 1st, 3rd, 5th, 7th, 9th, 11th, 13th, and 15th day, the wound area was photographed. The wound closure area was calculated by Image J. Wound contraction (%) was calculated using the equation: Wound contraction (%) = (area (1 day) − area (n day))/(area (0 day)) × 100%. The mice were executed under anesthesia and the regenerated skin in the wound area was cut and fixed with 4% paraformaldehyde fixation solution.

#### 4.6.4. Histological Analysis

In order to evaluate the epidermal regeneration and inflammation in the wound area, skin tissue samples were routinely processed and stained with hematoxylin and eosin (H&E). Masson trichrome stain was used for evaluation of collagen content at the wound area. All slices were analyzed and photographed by microscope (Aperio CS2, San Diego, CA, USA). Wound healing criteria were evaluated including reepithelization, granulation tissue formation, inflammation, and angiogenesis.

#### 4.6.5. Immunohistochemistry

The tissue sections were cut into slices, deparaffinized, rehydrated, antigen-retrieved, stained, and then the fixed sections were stained with anti-CD31 (Abcam, Cambridge, UK), anti-c-Kit (Abcam), anti-HSP47 (Proteintech, Wuhan, China), and anti-α-SMA (Proteintech). All slices were analyzed and photographed by microscope (Leica, Germany). Microscopic angiogenesis was evaluated.

### 4.7. Statistical Analysis

Data were revealed as mean ± SD. One-way analysis of variance, followed by a post hoc test, was utilized to assess the differences among the experimental groups. The difference was significant if *p* < 0.05 using Prism version 8 (GraphPad Software, Inc., San Diego, CA, USA).

## 5. Conclusions

In summary, we develop a hydrogel dressing with rapid shape adaptability, fast self-healing, great tissue adhesion, antioxidant activity, good antibacterial properties, and biocompatibility that can facilitate promising wound healing and skin tissue regeneration. The hydrogel dressing was obtained by using sodium periodate to cross-link CS-GA. The functional hydrogel simultaneously exerted multifunctional bioactivities including antibacterial and anti-inflammatory effects, accelerated collagen deposition, showed proliferative effects of fibroblasts, and a proangiogenic effect by the release of scolopin2, all of which facilitated the outstanding wound repair. In addition, the hydrogel, with a facile synthesis process, eximious physical properties, and good biological activity and biocompatibility, provides more possibilities for clinical applications.

## Data Availability

Data are contained within the article and Supplementary Materials.

## Supplementary Materials

The following supporting information can be downloaded at: https://www.mdpi.com/article/10.3390/ph15111422/s1, Figure S1: Wound condition of mice in the day 15 model group after pulling; Video S1: The injectability of hydrogel; Video S2: The spray ability of hydrogel.
