# Supplementary figures and images for: A Shape-Adaptive Gallic Acid Driven Multifunctional Adhesive Hydrogel Loaded with Scolopin2 for Wound Repair
Source: Pharmaceuticals (Basel). 2022 Nov 17;15(11):1422. doi: 10.3390/ph15111422 (PMC9695609; doi:10.3390/ph15111422)

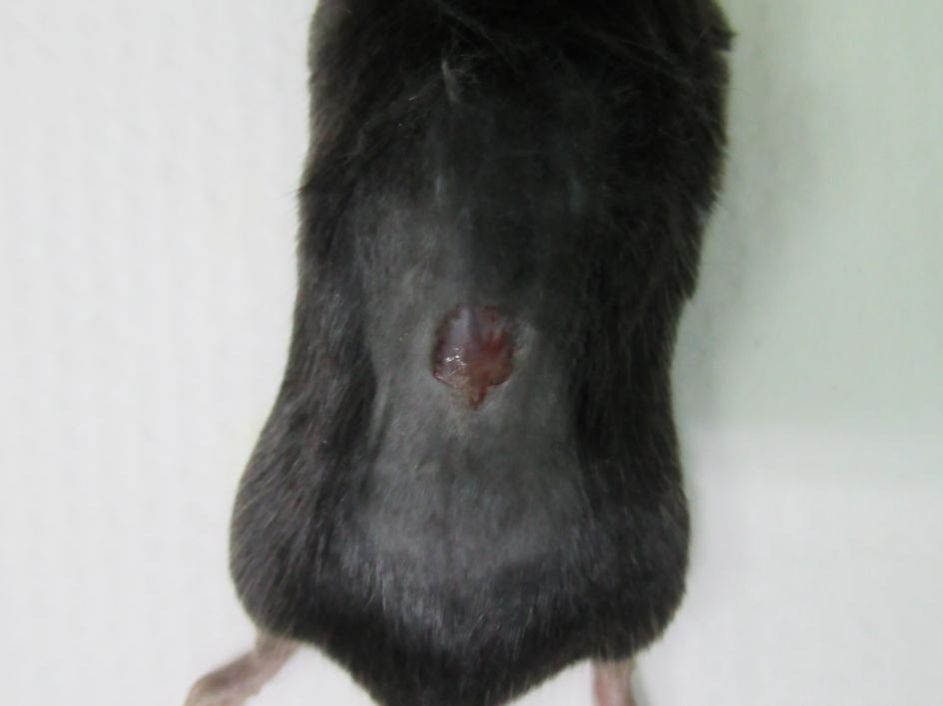

Supplement: Supplementary file 1 [file pharmaceuticals-15-01422-s001.zip › Figure S1.tif]
